# Canadian Nationwide Survey on Pediatric Malnutrition Management in Tertiary Hospitals

**DOI:** 10.3390/nu13082635

**Published:** 2021-07-30

**Authors:** Koen Huysentruyt, Kim Brunet-Wood, Robert Bandsma, Leah Gramlich, Bonnie Fleming-Carroll, Brenda Hotson, Rebecca Byers, Heather Lovelace, Rabin Persad, Daina Kalnins, Andrea Martinez, Valerie Marchand, Mélanie Vachon, Jessie M. Hulst, on behalf of the Canadian Malnutrition Task Force—Pediatric Working Group

**Affiliations:** 1 Division of Paediatric Gastroenterology, Hepatology and Nutrition, Department of Paediatrics, The Hospital for Sick Children, Toronto, ON M5G 1X8, Canada; robert.bandsma@sickkids.ca (R.B.); jessie.hulst@sickkids.ca (J.M.H.); 2 Department of Pediatric Gastroenterology, Universitair Ziekenhuis Brussel, Vrije Universiteit Brussel (VUB), 1090 Brussels, Belgium; 3 Canadian Malnutrition Task Force, Canadian Nutrition Society, Ottawa, ON K1C 6A8, Canada; kimbrunetwood8@gmail.com; 4 Department of Nutritional Sciences, University of Toronto, Toronto, ON M5S 1A8, Canada; 5 Division of Gastroenterology, University of Alberta, Edmonton, AB T6G 2G3, Canada; leah.gramlich@ualberta.ca; 6 SickKids Learning Institute, The Hospital for Sick Children, Toronto, ON M5G 1X8, Canada; bonnie.fleming-carroll@sickkids.ca; 7 Health Sciences Centre, Winnipeg, MB R3A 1R9, Canada; BHOTSON@wrha.mb.ca; 8 Canadian Nutrition Society, Ottawa, ON K1C 6A8, Canada; cara@cns-scn.ca; 9 Nutritional Services, BC Children’s Hospital, Vancouver, BC V6H 3N1, Canada; hlovelace@cw.bc.ca; 10 Division of Pediatric Gastroenterology, Hepatology and Nutrition, University of Alberta, Edmonton, AB T6G 1C9, Canada; Rabin.Persad@albertahealthservices.ca; 11 Department of Clinical Dietetics, The Hospital for Sick Children, Toronto, ON M5G 1X8, Canada; daina.kalnins@sickkids.ca; 12 Division of Paediatric Gastroenterology, Hepatology and Nutrition, Department of Paediatrics, IWK Health Centre, University of Dalhousie, Halifax, NS B3K 6R8, Canada; Andrea.Martinez@iwk.nshealth.ca; 13 Division of Paediatric Gastroenterology, Hepatology and Nutrition, Department of Paediatrics, Ste-Justine UHC, University of Montreal, Montreal, QC H3T 1C5, Canada; val.marchand@sympatico.ca; 14 Department of Clinical Nutrition, Quebec University Hospital, Quebec, QC G1R 2J6, Canada; melanie.vachon@mail.chudequebec.ca

**Keywords:** child, disease-related malnutrition, nutritional assessment, nutritional screening

## Abstract

**Background:** Disease-associated malnutrition (DAM) is common in hospitalized children. This survey aimed to assess current in-hospital practices for clinical care of pediatric DAM in Canada. **Methods:** An electronic survey was sent to all 15 tertiary pediatric hospitals in Canada and addressed all pillars of malnutrition care: screening, assessment, treatment, monitoring and follow-up. **Results:** Responses of 120 health care professionals were used from all 15 hospitals; 57.5% were medical doctors (MDs), 26.7% registered dietitians (RDs) and 15.8% nurses (RNs). An overarching protocol for prevention, detection and intervention of pediatric malnutrition was present or “a work in progress”, according to 9.6% of respondents. Routine nutritional screening on admission was sometimes or always performed, according to 58.8%, although the modality differed among hospitals and profession. For children with poor nutritional status, lack of nutritional follow-up after discharge was reported by 48.5%. **Conclusions:** The presence of a standardized protocol for the clinical assessment and management of DAM is uncommon in pediatric tertiary care hospitals in Canada. Routine nutritional screening upon admission has not been widely adopted. Moreover, ongoing nutritional care of malnourished children after discharge seems cumbersome. These findings call for the adoption and implementation of a uniform clinical care pathway for malnutrition among pediatric hospitals.

## 1. Introduction

Recent studies reported that disease-related malnutrition (DAM), which can be defined as malnutrition in the context of acute and/or chronic disease, is highly prevalent in hospitalized children in North America [1,2,3]. Nine percent of the children had a body mass index (BMI) or weight for height (WFH) below −2 SD on admission [1,2], which is in close agreement with figures reported in Europe [4]. The use of WFH < −2 SD or BMI < −2 SD (or WFA < −2 SD for infants) as indicators of malnutrition stems from recommendations made by the World Health Organization to screen for children with malnutrition. In the context of DAM, experts agree that it takes more than a single below-normal anthropometric measure to classify a child as being malnourished, due to the multifactorial etiology of DAM. Less agreement is found on how to accurately define pediatric DAM in clinical practice. The conceptual framework by Mehta et al., using five key domains (anthropometric parameters, growth, chronicity, etiology and outcomes) [5] has gained acceptance, but less is known about how this definition is translated into clinical practice. A large international survey involving almost 700 pediatric gastroenterologists and pediatric registered dietitians (RDs) from Europe and Australia identified ongoing weight loss, increased energy or nutrient losses, increased requirements, low intake and a high-risk condition as the most valued clinical indicators of DAM in daily clinical practice [6]. Although there are recommendations available from the WHO and Academy of Nutrition and Dietetics/ASPEN, literature on North American practices of assessment and management protocols for pediatric DAM is lacking. Furthermore, available survey literature from other continents mainly focuses on what happens during hospitalization [6,7,8]. Therefore, we aimed to survey all tertiary pediatric hospitals in Canada to address all pillars of DAM care in hospitalized children: prevention, screening and assessment, treatment as well as post-hospitalization follow-up. We also aimed to address education and training related to DAM. The survey was issued by the Canadian Malnutrition Task Force (CMTF) Pediatric Working Group and is intended to clarify how the current state of in-hospital practice relates to current guidelines and to serve as a base for the development of an overarching, nationwide consensus protocol. More specifically, the following aims were put forward:

1. To survey current in-hospital practices for the screening, assessment and management of pediatric DAM in Canadian tertiary hospitals.
2. To obtain knowledge about current discharge practices and follow-up protocols for the management of DAM.
3. To inquire about further education and training desires of tertiary care-level pediatric health care providers (registered dietitians (RDs), registered nurses (RNs) and medical doctors (MDs)) caring for malnourished children.

## 2. Materials and Methods

### 2.1. Survey Development and Distribution

A draft questionnaire was conceived by a core group of the CMTF Pediatric Working Group. This draft was based on a review of the literature on pediatric nutritional screening and assessment. The questionnaire included a total of 40 questions, of which five questions were available to RDs only. The draft was piloted by the other members of the CMTF Pediatric Working Group and issues around clarity of questions were discussed until final agreement was met. The survey was approved by the Human Research and Ethics Board (HREB) of the University of Alberta (Pro00089906) and consent was obtained from all participants. To ensure that complete insight into the current state of in-hospital practices was obtained, questions were divided into different sections: “administrative” questions (e.g., profession and hospital affiliation), prevention, nutritional screening and assessment methods and protocols, treatment, follow-up at discharge and education and training. The original English questionnaire was translated into French by native Francophone group members and distributed in both languages. A list of all available Canadian tertiary pediatric hospitals (*n* = 15) was drafted to identify all target hospitals. The survey was sent electronically in April 2019 to the lead RDs of every hospital who served as contact persons for this study. The lead RDs were identified through personal correspondence and were asked to distribute the survey to nurses (RNs) and medical doctors (MDs) working in non-acute pediatric specialties (i.e., excluding emergency medicine, NICU and PICU services) with the aim of collecting as many answers as possible from within each institution. A deliberate choice was made not to include critical care settings, as the nutritional management protocols are likely to differ substantially from those on regular ward services. The contact RDs were asked to complete the survey on behalf of RDs in their facility, but in four centers it was seen fit by the lead RD to also include the opinion of other RDs; this was done at their discretion. Targeted reminders were sent until responses from all hospitals were obtained to ensure the generalizability of our results to the entire Canadian setting; the survey was closed for further response in October 2019. All targeted centers responded to the survey, but data on the response rates of the internal distribution in each hospital were not collected. An English version of the survey is available in Supplementary file S1.

### 2.2. Statistical Analysis

Statistical analysis was performed using R (version 3.6.2; R Foundation for Statistical Computing, Vienna, Austria, 2014) [9]. Descriptive statistics included means or medians for continuous variables and frequencies, modes (i.e., the most frequent answer) and percentages of categorical variables. Differences in proportions between groups were analyzed using χ²-test or Fisher’s exact test. Continuous variables were compared using a Wilcoxon Mann–Whitney test. Odds ratios and their 95% CIs were calculated based on a mixed logistic regression model using the response to one question and the profession as predictors and the response to the question of interest as the binary dependent variable; the hospital was used a random effect. Missing values were reported separately per question. A *p*-value of <0.05 was considered statistically significant.

## 3. Results

### 3.1. Characteristics of Participants and Participating Hospitals

A total of 147 medical professionals responded to the questionnaire. Of these, 27 (18.4%) respondents were removed from further analyses because they were either working in the outpatient service of the hospital only or involved in critical care pediatric specialty teams. Among the remaining 120 participants, 69 (57.5%) were MDs, 32 (26.7%) RDs and 19 (15.8%) RNs; participants were working in general pediatrics, surgery and various subspecialties. Both MDs and RNs were asked to self-score (min 1 to max 10) their interest and knowledge about clinical nutrition, and the median (Q1;Q3) scores for MDs (*N* = 51) were 9 (7;10) and 7 (6;10), respectively; for RN (*N* = 12) these were 6.5 (5;8) and 4.5 (3;6). All targeted hospitals were represented, with a minimum of one (CHUL Quebec), a median of 5.5 and a maximum of 34 (CHU Sainte-Justine) participants belonging to the same hospital (four participants did not disclose which hospital they belonged to). At least one RD responded from each hospital, while responses from MDs and RNs came from 9/15 hospitals. For three hospitals (CHUL Quebec, CHU Sherbrooke, Montreal Children), only responses from RDs were collected. The number of inpatient pediatric beds in the participating hospitals ranged from 42 to 307 (no significant difference in median number of beds between centers with only an RD responding vs. those with multiple professions represented; *p* = 0.201). The number of full-time equivalent RDs working in inpatient care ranged from a minimum of 1.5 to 21 (data available for 13/15 hospitals; no difference between RD-only centers and other centers, *p*-value 0.806).

### 3.2. Nutritional Screening and Assessment

**Figure 1:**
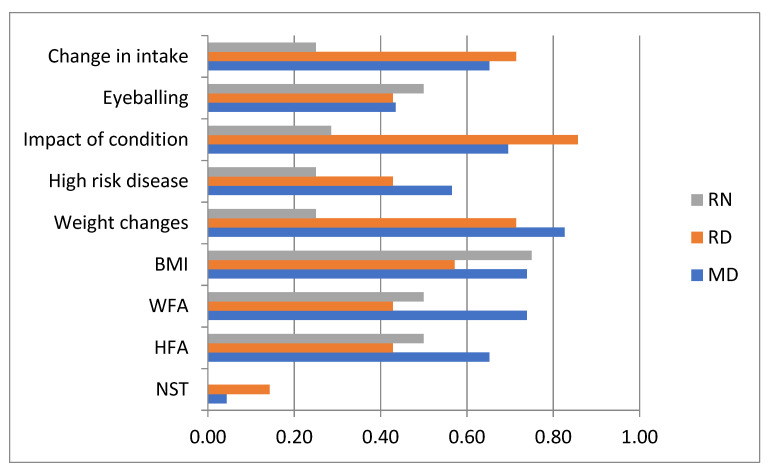
Nutritional screening practices in Canadian tertiary pediatric hospitals by health care profession. RN: registered nurses, RD: registered dietitians, MD: medical doctors, BMI: body mass index, WFA: weight for age, HFA: height for age, NST: nutrition screening tool; results expressed as a proportion of the respondents for this question (*n* = 59).

An overarching protocol for the prevention, detection and intervention for pediatric malnutrition that applies to all pediatric services was present or “a work in progress”, according to only 10/104 (9.6%) respondents, with at least one representative indicating the awareness of such a protocol in 7/15 centers. Routine nutritional screening on admission was “always” performed according to 15.7% (16/102) of the respondents, “sometimes” by 43.1 % and “never” by 10.8%, another 30.4% responded they were unaware of screening practices in their hospital (Fisher’s exact test, *p* = 0.009). The answers “always” and “never” coincided, however, in 3/15 hospitals. An overview of the different nutrition screening measures used in clinical practice is given in Figure 1. Most respondents (52/59) used a combination of multiple screening modalities, and only three (from different hospitals) responded that they used validated screening tools. The overall mode (45/59) regarding screening was “assessment of changes in weight (loss or slow weight gain)”, followed by “assess the impact of the current condition (chronicity and severity) on intake requirement”, which was considered by 40/59. These two screening methods were considered by the majority of the RDs and MDs, while no single screening method clearly stood out for the RNs. Patients or family were named by 87.5% (63/72) of the respondents as the party that raised nutrition issues to the medical staff (the question was not asked of participating RDs). Interestingly, 91.5% of the MDs also named the medical staff as this person, while only 46.2% of the RNs considered the medical staff (95% CI OR 0.02–0.39).

**Figure 2:**
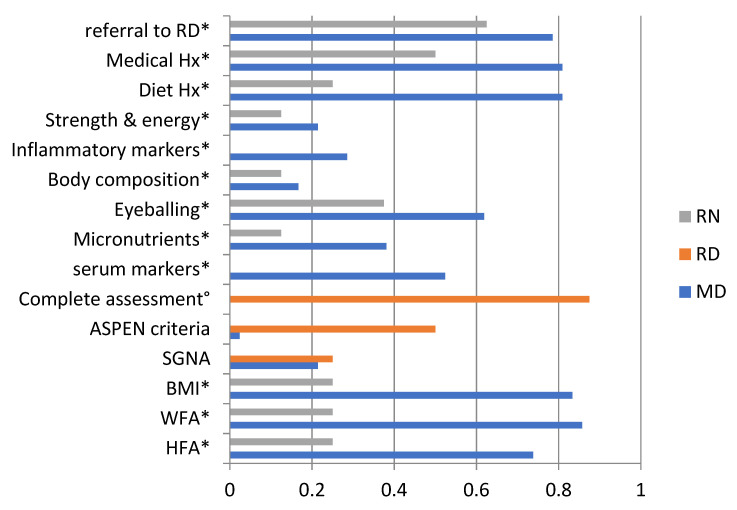
Approaches to nutritional assessment in Canadian tertiary pediatric hospitals by health care profession. RN: registered nurses, RD: registered dietitians, MD: medical doctors, BMI: body mass index, WFA: weight for age, HFA: height for age, SGNA: Subjective Global Nutritional Assessment, Strength and Energy: “Assess strength and energy levels”; * option only available for RN and MD; ° option only available to RD; results expressed as a proportion of the respondents for this question (*n* = 42), multiple answers were allowed.

The nutritional status of newly admitted children was “always” routinely assessed according to 18.6% (18/97) of the respondents, “sometimes” by 53.6 % and never by 6.2%; another 21.6% responded they were unaware of assessment practices in their hospitals (Fisher’s exact test, *p* = 0.003). The answers “always” and “never” coincided, however, in 4/15 hospitals. Respondents who were unaware of routine nutrition screening practices had significantly higher odds of also being unaware of nutritional assessment practices, while accounting for their profession (95% CI OR 8.7;218.0). An overview of the different approaches to nutrition assessment used in practice is given in Figure 2. Weight z-scores or centiles was the mode for MDs, with a response of 85.7% (36/42); the second and third most frequent assessment modalities were BMI (83.3%) and diet and medical history (both 81.0%). For RNs, the most preferred option was referral to an RD (5/8, 62.5%).

A protocol for measurement techniques, equipment and frequency was present for inpatients, according to 41.9% (39/93) of the respondents, although conflicting answers (yes and no) within a hospital were present for 8/15 hospitals. Having answered that there is a measuring protocol present was associated with significantly higher odds of answering “always” or “most of the times” to the question of whether a child was weighed within 24 h of admission (95% CI OR 2.3; 17.3) but not to answering “never” or “sometimes” to the use of invalid measuring techniques (use of a stretched tape measure or asking parents for a height; 95% CI OR 0.2; 2.5) while accounting for profession.

### 3.3. Nutritional Management

Surprisingly, 32.1% did not differentiate in food intake monitoring practice between the general population of admitted children and malnourished children specifically (no significant difference between professions, *p* = 0.858). The mode for monitoring of food intake was “no regular monitoring” (52/81, 54.2%) when the entire population of hospitalized children was considered and “calorie counts” (53/81, 65.4%) when only the malnourished children were considered. Having answered that there is adequate inpatient RD staffing to provide nutrition care in a timely fashion was borderline associated with significantly higher odds of selecting calorie counts for monitoring malnourished children (95% CI OR 0.99; 57.9), but not all children (*p* = 0.940). When asked to rank the most used nutritional intervention practice, optimization of a child’s oral intake was considered by the vast majority as the primary choice (86.4%, 70/81), while initiating enteral nutrition (EN) or parenteral nutrition (PN) was among the lowest ranked choices (50.6%). The initiation of oral nutrition supplement was most frequently ranked as second (64.2%), but considered as a first choice by only 4.9%. There was no significant difference in the distribution of the primary choice for first intervention across professions (*p* = 0.607), but the lowest ranked choice was significantly different across professions (EN/PN in 54.9% of MDs and 63.6% of RNs, Med Pass in 89.5% of RDs; *p* = 0.001).

### 3.4. Discharge Practices and Follow-Up Protocols

Information about discharge and follow-up practices is summarized in Table 1. Anthropometrics were acquired “always” or “most of the time” in all patients at discharge, according to 4/13 (30.8%) RNs and 20/51 (39.2%) MDs (*p* = 0.139), while only 1/19 RD (4.0%) agreed with this. Nutritional status information was routinely provided in the discharge letter, according to 16/52 (30.8%) MDs but only 1/19 (5.3%) RD, whereas 59.6% and 63.2%, respectively, believed this was done only if there was a poor nutritional status noted during admission (distribution of all responses in MD vs. RD: *p* = 0.025). The most used term to describe poor nutritional status was “failure to thrive” (39/51 MDs, 13/19 RDs), followed by “growth failure” (16/51 MDs, 6/19 RDs) and an ICD-10 code that specified malnutrition (13/51 MDs, 9/19 RDs). For children with a poor nutritional status, nutritional care was sometimes/never transferred to another professional for follow-up, according to 33/68 (48.5%, no difference across professions; *p* = 0.852). A lack of staff to refer to (MD: 35/43, RD: 14/18) and a low medical staff awareness of the role of nutrition in patient care (MD: 35/43, RD: 14/18) were both regarded as equally important barriers to adequate transfer of nutrition care. The odds of always transferring nutrition care were not significantly different, based on having identified a barrier to transferring nutrition care (95% CI OR 0.4; 3.0), while accounting for profession. When patients were referred, the primary choice for referral was most frequently to an outpatient RD (19/65, 29.2%), pediatrician or clinic (both 13/65, 20.0%).

### 3.5. Further Education and Training Desires

**Figure 3:**
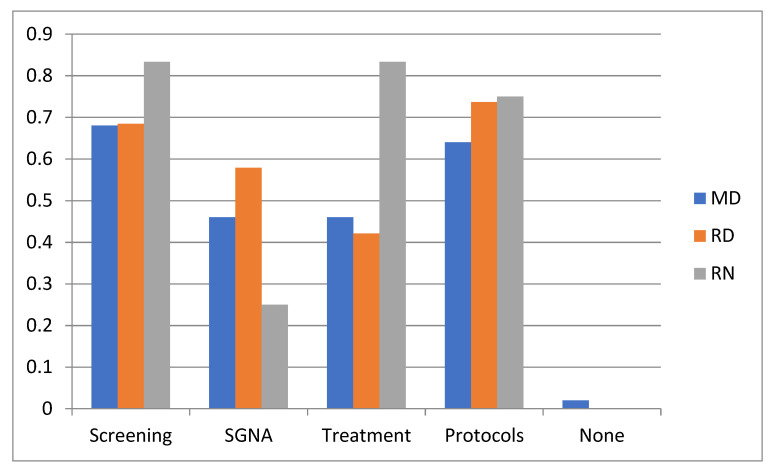
Interests in further nutrition-related education in Canadian tertiary hospitals by health care profession. RN: registered nurses, RD: registered dietitians; MD: medical doctors; SGNA: Subjective Global Nutritional Assessment; results expressed as a proportion of the respondents for this question (*n* = 42).

In an open-ended question inquiring about suggestions for the improvement of nutrition care, a request for clear protocols/screening systems (22/38, 57.9%), more resources (21/38, 55.3%) and training and increased awareness (11/38, 28.9%) were most frequently suggested. Figure 3 shows an overview of the topics that were indicated as most interesting for further education. Overall (*N* = 81), the most interest was given to education about screening and protocols, while education about the SGNA was less attractive to RNs as compared to RDs or MDs. The preferred learning modality was via E-learning or online modules (66/81, 81.5%), followed by courses or workshops in the own hospitals (64.2%). International conferences were the least preferred learning modality (4.9%).

## 4. Discussion

This is the first survey to investigate pediatric nutritional management in Canadian tertiary hospitals. We were able to obtain responses from every pediatric tertiary care center in Canada, ensuring generalizability of our results to the entire Canadian tertiary setting. Our survey demonstrated that systematic nutritional screening has not been widely adopted in these hospitals, and that the use of validated nutrition screening tools in Canadian tertiary pediatric centers is rather an exception in clinical practice. The lack of a standardized approach to pediatric nutritional assessment was apparent as answers differed not only among hospitals, but also between respondents from the same hospitals. Lastly, this study was the first to investigate nutrition discharge and follow-up practices for hospitalized children. Nutritional care of malnourished children was rarely transferred to another professional for follow-up according to almost half of the respondents.

Systematic screening was performed by only 15% of the respondents, although almost half reported to screen sometimes. Interestingly, the answers “always” and “never” coincided in one-fifth of the hospitals. This discrepancy could be explained by the lack of a written protocol in most of the centers, but perhaps also relates to the philosophical differences around what constitutes nutritional screening for hospitalized children. Performing nutritional screening has been advocated by the European Society for Pediatric Gastroenterology, Hepatology and Nutrition (ESPGHAN) and the American Society for Parenteral and Enteral Nutrition (ASPEN) [5,10]. Different practices that constitute nutritional screening were noted in our survey, which is in line with previous reports [7]. A survey among Belgian pediatricians showed that only a small minority of the centers was using validated nutritional screening tools, while more than half of the respondents did indicate they performed some kind of nutritional screening [7]. Similar findings were reported in a South Korean survey [8]. While some favor the advantage of validated screening tools that are able to filter out patients at risk of nutritional deterioration during hospitalization and favor their simplicity [11], others give preference to axiological methods as they find them more pragmatic [5,12]. The importance of having a protocol was also reflected by the fact that respondents with a written protocol for measurement techniques, equipment and frequency were more likely to find that children get weighed within 24 h of admission in their center.

Different terms were used by our respondents to describe the presence of malnutrition in discharge communications, with the generic term “failure to thrive” (FTT) being the most popular term. FTT is a generic concept and most used in the context of faltering growth early in childhood, and many criteria have been to define it. Olsen et al. compared seven commonly used criteria to what they considered a true diagnosis of FTT—BMI <5th percentile and a conditional weight gain <5th percentile—and found that different criteria identified different children [13]. In their opinion, no single criterion was able to reliably identify growth delay. ICD-10 codes that specified malnutrition were the least popular choice to describe malnutrition. An American retrospective study using these ICD codes concluded that the exclusive use of these codes might contribute to the underdiagnosis of pediatric malnutrition [14].

Standard monitoring practices of food intake differed on whether the respondents were dealing with malnourished children or not. Surprisingly, most of the respondents of each profession agreed that there is no regular monitoring of food intake when considering all inpatients. Calorie counting, a highly labor-intensive process, was reserved for the monitoring of malnourished children by most of the respondents. A possible influence on this approach of dietetic staffing was found. Becker et al. underlined the importance of routinely estimating the adequacy of protein/energy intake in their ASPEN consensus statement on clinical indicators recommended for the identification of pediatric malnutrition [15]. They did not specify, however, which method to use except that “food/nutrient intake details can be obtained by history and/or by direct observation of food and/or nutrients consumed.” Previous pediatric studies on food intake monitoring are scarce. A previous Canadian study on food choices of hospitalized children suggested that questionable food choices were made via a centralized computer ordering system [16], although the primary concern was for obesity in this study. Carter et al. found children having unique barriers compared to adults in a study where they examined barriers to oral intake in hospitalized children. Children are more affected by the quality of food and report being hungry while the adults reported missing meals due to tests or inability to open packaging. The traditional hospital food model did not meet the needs of the pediatric patients studied [17]. From studies in adult populations it is known that insufficient nutritional intake during hospitalization is highly prevalent and can lead to and aggravate hospital malnutrition and affect outcome [18,19,20,21,22,23]. Adult studies have reported that calorie counts are commonly used to monitor nutritional intake but are variably reported, as we found in our survey [24,25]. Moreover, these studies showed that the results of calorie counting, especially when done on flow sheets, were not necessarily used to guide interventions [24,25]. Studies performed by CMTF among adult hospitalized patients in Canada also showed that routine food monitoring practices were not highly prevalent, but that after the implementation of the Integrated Nutritional Pathway for Acute Care (INPAC), which includes standard food intake monitoring, its practice was increased [26]. The optimization of a child’s oral intake was the most preferred primary intervention among all HCP groups, which is in line with international recommendations [27].

The organization of nutritional follow-up post-discharge seemed to be problematic for a sizable portion of our respondents. Almost half of them responded that even for children with a poor nutritional status during hospitalization, nutritional care was at best sometimes transferred to another professional for follow-up. Reasons identified for this were a lack of staff to refer to and limited medical staff awareness of the role of nutrition. This is remarkable as the median length of hospital stay of children in a mixed population of Canadian hospitalized children was only 3–5 days [2,3]. It is highly unlikely that nutritional issues, especially in malnourished children, are resolved in such a short time span. As outpatient pediatric RDs were the primary choice to refer to, a scarcity of these health care professionals, at least in certain areas of the country, might explain this. This hypothesis is strengthened by the fact that more than half of our respondents suggested that more resources could improve nutrition care in Canadian tertiary hospitals. Exploration of the additional info provided by the respondents indicated that the perceived lack of resources almost univocally constituted a perceived lack of dieticians or HCP adequately trained in pediatric nutrition.

We also questioned the interest of our respondents in further nutrition-focused training and found that the most interest was given to education about screening and nutritional assessment and management protocols. The Pediatric Working Group of the Canadian Malnutrition Task Force (CMTF) is currently undertaking an effort to develop a clinical practice pathway for prevention, detection and treatment of DAM that is supported by key stakeholders across the country. This clinical practice pathway will aim to standardize the approach to malnutrition screening, prevention, assessment and treatment across Canadian pediatric hospitals.

The major strength of our study is that it is representative of tertiary care-level pediatric centers in Canada, as we received responses from all centers across the country. Some of the respondents from the same centers, however, provided conflicting answers, which could indicate that they are more aware of what happened on their own wards than of what happened in the entire hospital. This could also indicate a lack of organizational policies and protocols related to assessment and management of DAM in their hospitals. Furthermore, our study was the first to inquire about post-discharge practices, revealing a major lacuna in nutritional care for malnourished children in the hospital. A limitation of this study is the fact that no exact response rate could be calculated, since it was unclear how many different health care professionals the survey was distributed to within each hospital. In addition, the different medical professions were not equally represented among the hospitals, which could have affected the results. Although similarities in some of the issues were found between this survey and data from other countries [7,8], the results of this national survey might not necessarily be applicable to other countries. For example, the clinical responsibilities of RDs can differ widely among countries. In Canada, RDs are frequently involved in the preparation of prescriptions of total parenteral nutrition and perform clinical examinations, while this is less frequent in, for example, different European countries. Lastly, the results of this survey apply only to tertiary care hospitals and the practices on pediatric wards of general hospitals may be different, so we cannot extrapolate our results to all pediatric inpatient care.

In conclusion, our survey demonstrated that systematic nutritional screening and especially the use of validated pediatric nutrition screening tools have not been widely adopted in Canadian tertiary pediatric hospitals. A lack of uniformity in the approach to pediatric nutritional assessment was apparent across and within different hospitals. Lastly, our data suggest that ongoing nutritional care of malnourished children after discharge from hospital is not well established.

## Figures and Tables

**Table 1:** Nutrition discharge and follow-up practices in Canadian tertiary pediatric hospitals.

D/C or Follow-Up Practice	N * (%)
Info on nutritional status in D/C summary:	
Yes	20 (23.8)
Only if malnourished	50 (59.5)
No/don’t know	10 (11.9)
Other	4 (4.8)
Weight and height measured at D/C:	
Always	6 (7.2)
Mostly	19 (22.9)
Sometimes	29 (34.9)
Never	10 (12.0)
Other	19 (22.9)
Terms describing poor nutritional status in D/C summary °:	
Failure to thrive	52 (74.3)
Growth failure	22 (31.4)
ICD-10 code	22 (31.4)
Not common/not applicable	5 (7.1)
Other	11 (15.7)
Post-D/C transfer of nutrition care of malnourished child:	
Always	27 (39.7)
Sometimes	32 (47.1)
Never	1 (1.5)
Don’t know	8 (11.8)

D/C: discharge; * Answers of all professions combined; ° multiple answers possible and only RD/MD included.

## Data Availability

Not applicable.

## Supplementary Materials

The following are available online at https://www.mdpi.com/article/10.3390/nu13082635/s1, Supplementary file S1: English version of the original survey.
