# Supplementary material for: Canadian Nationwide Survey on Pediatric Malnutrition Management in Tertiary Hospitals
Source: Nutrients. 2021 Jul 30;13(8):2635. doi: 10.3390/nu13082635 (PMC8397996; doi:10.3390/nu13082635)
Supplement: Supplementary file 1 [file nutrients-13-02635-s001.zip › nutrients-1285281-supplementary.pdf]

Supplementary file S1. Original questionnaire

1. In which Children's Hospital do you work?

Alberta Children's Hospital

British Columbia Children's Hospital

Centre hospitalier universitaire de Québec, Centre mère enfant Soleil

Centre hospitalier universitaire Sainte-Justine

Centre hospitalier universitaire de Sherbrooke-Hôpital de Fleurimont

Children's Hospital of Eastern Ontario

Children's Hospital at London Health Sciences Centre

The Children's Hospital of Winnipeg

The Hospital for Sick Children

IWK Health Centre

Janeway Children's Health and Rehabilitation Centre

McMaster Children's Hospital

Montreal Children's Hospital

Saskatchewan Children's Hospital

Stollery Children's Hospital

2. What is your profession?

General pediatrician (MD)

Pediatric medical specialist

Dietitian (RD)

Pediatric nurse (RN)

Medical/Nursing Survey

3. What type of hospital unit are you currently working in? (Select only one. If you work at multiple units, select the PRIMARY unit.)

Cardiology

Endocrinology

Gastroenterology

General Medicine

Genetics

Nephrology

Neurology

Oncology

Respiratory

Surgery

Transplant

Other (please specify)

NUTRITIONAL CARE IN YOUR HOSPITAL – PREVENTION - DETECTION

4. Does the department of pediatrics have an overarching protocol or guideline outlining prevention, detection and intervention for pediatric malnutrition that applies to all pediatric services?

Yes

Work in progress

No

Do not know

Other (please specify)

5. (If yes) Which of the following does the protocol/guideline cover? (Select all that apply)

Nutritional screening

Nutritional assessment

Nutritional intervention

Scope of responsibility for dietitians

6. Is your inpatient RD staffing adequate to provide nutrition care in a timely fashion?

Yes

No

Don't know

7. (If yes) Do the dietitians take part in one or more nursing, medical or interdisciplinary rounds on the unit?

Yes, it is a regular activity

Yes, but only on specific units

No

Don't know

NUTRITIONAL SCREENING & ASSESSMENT IN HOSPITALIZED CHILDREN

8. Indicate the frequency with which nutrition screening for undernutrition in pediatric patients occurs upon admission to hospital?

Always

Sometimes

Never

Don't know

If Sometimes, please specify:

9. Which of the following approaches do you use to routinely screen for undernutrition in pediatric patients on admission to the hospital? (Select all that apply)

Validated malnutrition risk screening tools (e.g. StrongKids, PNST, PYMS, STAMP)

Height for age percentiles or Z scores
Weight for age percentiles or Z scores
BMI or weight for height percentiles or Z scores
Assess changes in weight (loss or slow weight gain)
Classify them according to their underlying condition (e.g. high nutritional risk disease)
Assess the impact of the current medical condition on intake, and/or requirements
Visual inspection (eye-balling)
Assess changes in normal/usual dietary intake
Other (please specify)

10. Which validated nutritional risk screening tool do you use?
PYMS
STAMP
STRONGkids
PeDiSMART
PNST
Other (please specify)

11. How is the malnutrition screen administered?
Electronic directly in electronic medical record (EMR)
Paper form
Other (please specify)

12. Who is primarily responsible for performing the nutritional risk screening tool?
Attending pediatrician
Attending resident/fellow
Nurse admitting the child
Dietitian
Other (please specify)

13. Who is primarily responsible for interpreting the score of the nutritional risk screening tool and arrange the next steps advised by the tool?
Most responsible physician
Most responsible fellow/resident
Nurse taking care of the child
Dietitian
Other (please specify)

14. Indicate the frequency with which <u>nutritional status</u> is routinely assessed on admission to hospital?
Always

Sometimes
Never
Don’t know

If Sometimes, please specify:

15. Which of the following approaches do you use to assess nutritional status? (Select all that apply)
Height for age percentiles or Z scores
Weight for age percentiles or Z scores
BMI or weight for height (WFH) percentiles or Z scores
Subjective Global Nutrition assessment (SGNA)
Academy of Nutrition and Dietetics (Academy) and ASPEN Consensus Malnutrition Characteristics for Pediatric Malnutrition
Measure blood nutritional markers (e.g. albumin, prealbumin)
Measure blood micronutrient status (vitamins and minerals)
Visual inspection (eye-balling)
Measure body composition (e.g. skin folds, MUAC)
Measure inflammatory markers (e.g. CRP)
Assess strength and energy levels
Diet history
Medical history
Refer to a dietitian to assess at risk children
Other (please specify)

16. Who raises nutrition issues to the medical team? (Select all that apply)
Medical staff
Nursing staff
Nutrition staff
Patient and/or Family

17. Does your facility have a protocol/policy that outlines growth measurement techniques, equipment and frequency for weight, height and head circumference measures for inpatients?
Yes
No
Don’t know

18. How often is the weight and height of children measured within 24 hours after admission to the hospital (or a few days in advance when the admission was planned for a procedure)?

Always
Most of the times
Sometimes
Never
Don't know

19. Who is primarily responsible for the interpretation of weight and height measurements, e.g. interpretation of growth and assessing the presence of a poor nutritional status?
Most responsible physician
Most responsible resident/fellow
Nurse taking care of the child
Dietitian
Other (please specify)

20. What equipment is used <u>routinely</u> to measure length or height in admitted infants/children? (Select all that apply)
Calibrated stadiometer or length boards
Tape measure
Knee height caliper
Ask parent for last measurement done at home
Height devices attached to scales
Other (please specify)

21. Are nutritional problems of admitted patients routinely taken into account on unit rounds?
Yes
No
Don't know

TREATMENT

22. If a child is identified as being at <u>nutrition risk on admission</u>, do you have standard practices outlined on next steps?
Yes
No
We do not screen

23. (If yes) What is the next step when a child is identified?
Referral to dietitian for assessment and nutritional advice
Complete a Subjective global nutritional assessment (SGNA)
Physician orders oral nutrition supplements

Physician orders nutrition support (enteral or parenteral nutrition)
Other (please specify)

24. If a child is identified as being malnourished, please rank the following choices in order of priority of usual practice. (1 = most common; 4 = least common)
Patients oral intake is optimized; example, high protein high calorie diet with likes/dislikes considered
Oral nutrition supplements are provided
Small amounts of nutrition supplements are provided at regular intervals (often called" Med pass")
Nutrition support (enteral or parenteral nutrition) is instituted

25. Are all patients regularly monitored to determine if they are meeting their food intake requirements? (Select all that apply)
They are not regularly monitored
Meal time audits
Calorie counts
Nurses chart % of meal eaten for each meal
Other (please specify)

26. Are malnourished patients regularly monitored to determine if they are meeting their food intake requirements? (Select all that apply)
They are not regularly monitored
Meal time audits
Calorie counts
Nurses chart % of meal eaten for each meal
Other (please specify)

27. Please review the following reasons that patients may not eat on your hospital unit and indicate the importance of each using the following rating system (Rarely a problem, Sometimes a problem, Commonly a problem, Don't Know)
Tray delivery is not coordinated between Food Services and Nursing
Appearance, taste, or aroma of food is poor
The meals are not appropriate for children
Food/fluid temperature is inappropriate
Patients are not given enough time to eat
Patient meals are interrupted by procedures or medical care
Patients' pain and symptoms are not well managed
Not able to give sufficient assistance with eating
Foods are not offered often enough

NUTRITIONAL FOLLOW-UP AT DISCHARGE

28. Are the weights/heights of children measured at discharge from the hospital? (Select the most applicable response.)

Always
Most of the times
Sometimes - Only when the length of stay was >5 days
Sometimes - Depends on child's age
Never
Other (please specify)

29. Is information about the nutritional status provided in the discharge summary? (Select the most applicable response.)

Yes, growth is commented on routinely including the difference between admission and discharge weight/height/or length and what would be expected.
Yes, and if malnutrition is present, it is included
No
No, not routinely, only if nutritional status was a problem noted during admission
Not sure
Other (please specify)

30. When a child is identified with poor nutritional status, which of the following terms do you record in your discharge summary? (Select all that apply)

Malnutrition or severe malnutrition based on ICD-10 codes
Failure to thrive
Growth failure
It is not a common problem, so don't have an opportunity to comment on it
Not applicable
Other (please specify)

31. When a child with malnutrition or poor nutritional status is discharged from the hospital, is the nutritional care transferred to another health care professional for follow-up?

Always
Sometimes
Never
Don't know
Other (please specify)

32. (If yes) Please order the following options, based on the frequency with which referrals are sent (1 = the most).

Patient seen by multidisciplinary team in ambulatory clinic

Dietitian in hospital continues care
Dietitian outpatient clinic
Specialist Pediatrician
Family doctor in primary care
Other (please specify)

33. What, if any, barriers do you experience for the adequate transfer of nutritional care for malnourished children after discharge from the hospital? (Select all that apply)
Lack of staff to refer to
Low staff awareness on the role of nutrition on patient care
Other (please specify)

34. How satisfied are you with the level of nutritional care for pediatric inpatients on a scale from 1 to 10, where 10 = is most satisfied and 1 = least satisfied?
1-10

35. What suggestions do you have for improvement of nutritional care?
(Open-ended question)

EDUCATION AND TRAINING

36. Rate your knowledge of the treatment of malnourished patients on a scale from 1 to 10, where 1 = inadequate knowledge and 10 = very good knowledge.
1-10

37. How interested are you in the treatment of malnourished patients on a scale from 1 to 10, where 1 = not interested and 10 = very interested?
1-10
38. On which topics, would you be interested in more education or training? (Select all that apply)
Education about nutritional screening
Education about Subjective Global Nutrition Assessment (SGNA)
Education about treatment of malnutrition
Education about nutrition protocols
None
Other (please specify)

39. What delivery format of training would you prefer? (Select all that apply)
E-learning or online modules
Online information
Course or workshop in your own hospital
Course or workshop in the region

Course or workshop on a national level
Course or workshop on an international level
Other (please specify)

40. Please provide further comments and/or questions as you wish.
(Open-ended question)
